# Evaluation of Two Models of Non-Penetrating Captive Bolt Devices for On-Farm Euthanasia of Turkeys

**DOI:** 10.3390/ani8030042

**Published:** 2018-03-20

**Authors:** Caitlin R. Woolcott, Stephanie Torrey, Patricia V. Turner, Lilia Serpa, Karen Schwean-Lardner, Tina M. Widowski

**Affiliations:** 1 Department of Animal Biosciences, University of Guelph, Guelph, ON N1G 2W1, Canada; caitwoolcott@gmail.com (C.R.W.); storrey@uoguelph.ca (S.T.); lilia.serpa20@gmail.com (L.S.); 2 The Campbell Centre for the Study of Animal Welfare, University of Guelph, Guelph, ON N1G 2W1, Canada; pvturner@uoguelph.ca; 3 Department of Pathobiology, University of Guelph, Guelph, ON N1G 2W1, Canada; 4 Department of Animal and Poultry Science, University of Saskatchewan, Saskatoon, SK S7N 5A8, Canada; karen.schwean@usask.ca

**Keywords:** animal welfare, euthanasia, turkey, insensibility, brain death

## Abstract

**Simple Summary:**

Animal care guidelines for livestock and poultry require farms to have euthanasia plans in place for birds that are sick, injured, or unable to access feed and water. Killing methods considered to be humane are those that induce rapid insensibility (stun) and result in brain death leading to irreversible respiratory and cardiac arrest. Therefore, the evaluation of the effectiveness of a killing method generally focuses on measures of insensibility and brain death. Non-penetrating captive bolt devices are intended to deliver sufficient force and energy to the head to result in immediate insensibility and brain death without penetrating the skin. We evaluated the effectiveness of two models of non-penetrating captive bolt devices when applied by stock people to different sizes and ages of turkeys, using signs of insensibility corroborated by ante- and post- mortem evaluation of brain damage. Both non-penetrating captive bolt devices used in this study were found to be highly effective at inducing immediate insensibility and would be appropriate for on-farm euthanasia of turkeys of various ages and size.

**Abstract:**

On-farm euthanasia is a critical welfare issue in the poultry industry and can be particularly difficult to perform on mature turkeys due to their size. We evaluated the efficacy of two commercially available non-penetrating captive bolt devices, the Zephyr-EXL and the Turkey Euthanasia Device (TED), on 253 turkeys at three stages of production: 4–5, 10, and 15–20 weeks of age. Effectiveness of each device was measured using both ante- and post-mortem measures. Application of the Zephyr-EXL resulted in a greater success rate (immediate abolishment of brainstem reflexes) compared to the TED (97.6% vs. 89.3%, *p* = 0.0145). Times to last movement (*p* = 0.102) and cardiac arrest (*p* = 0.164) did not differ between devices. Ante- and post-mortem measures of trauma and hemorrhage were highly correlated. Skull fractures and gross subdural hemorrhage (SDH) were present in 100% of birds euthanized with both the Zephyr-EXL and TED devices. Gross SDH scores were greater in birds killed with the Zephyr-EXL than the TED (*p* < 0.001). Microscopic SDH scores indicated moderate to severe hemorrhage in 92% of turkeys for the Zephyr-EXL and 96% of turkeys for the TED, with no difference between devices (*p* = 0.844). Overall, both devices were highly effective inducing immediate insensibility through traumatic brain injury and are reliable, single-step methods for on-farm euthanasia of turkeys.

## 1. Introduction

Animal care guidelines for livestock and poultry require farms to have euthanasia plans that include appropriate methods for birds that are sick, injured, or unable to access feed and water [1,2]. Euthanasia is defined as the humane killing of an individual animal in a way that minimizes pain and distress [3], with pain defined as an “unpleasant sensory or emotional experience” [4]. Killing methods considered to be humane are those that induce rapid insensibility (stun) and cause brain death that leads to respiratory and cardiac arrest [3]. Therefore, the evaluation of the efficacy of a killing method generally focuses on measures of insensibility and brain death.

Electroencephalography (EEG) is often considered the gold standard for determining loss of consciousness in humans as it directly measures electrical brain activity [5,6,7,8]. EEG recording in animals is only practical in a research setting at this point and other measures, such as loss of voluntary brainstem and spinal reflexes, and cessation of rhythmic breathing, are used to infer loss of sensibility. EEG recordings have been used to validate some of these measures in poultry. Loss of jaw and neck tone in turkeys and layer hens [7] and loss of posture in broiler chickens [9,10] have been confirmed as comparable indicators of loss of sensibility through studies using EEG analysis. Loss of pupillary light reflex has been confirmed as a reliable measure of brain death in turkeys and laying hens [7]. Nictitating membrane reflex is often also used as a measure of insensibility in poultry [11,12,13] and is considered to be a conservative measure of brain death [7]. The cessation of convulsions has been correlated with the time of isoelectric EEG measurements [14,15] and has been used as a proximate measure of brain death [9,16,17]. Cardiac arrest is often used as a measure of clinical death. However, the heart can continue to beat irregularly well after brain death has occurred [16,18].

For some killing methods, post-mortem measures of brain injury have been used for indirectly evaluating efficacy in poultry [19,20], swine [21,22,23,24], and lambs [25]. Physical methods of euthanasia (e.g., blunt force trauma, penetrating captive bolt device, cervical dislocation) are used to damage regions of the brain responsible for vital function through the application of a mechanical force that results in fatal traumatic brain injury. Traumatic brain injury (TBI) results from mechanical disruption through contusion, hemorrhage, and shearing of neurons [26]. Assessment of TBI can include macroscopic and microscopic examination and scoring of subdural and parenchymal hemorrhage [19,27], specifically in the areas of the brain responsible for consciousness and vital function (i.e., cerebral cortex and brainstem) [25,28]. An absence of hemorrhage in the brain has been associated with a lower stun efficiency; whereas moderate to marked hemorrhage has been found to be associated with immediate insensibility [21,22,29]. The severity of skull fracture is another measure used to assess the severity of TBI in both humans [30] and in animal studies [19,21,22,23,25,31]. Skull bone fracture in humans presenting with TBI from a blunt force is associated with higher mortality [30]. Cases of fatal head trauma in humans have also been associated with spinal cord injuries in the cervical region of the spinal column [32].

Cervical dislocation and blunt force trauma are common physical methods used on-farm because of their low cost, ease of use, and practicality [33]. Cervical dislocation is considered to be acceptable for poultry if it does not cause cervical crushing but is generally restricted to smaller birds (<3 kg) [1]. This poses a problem with turkeys because they quickly surpass the maximum weight requirement (<3 kg) for cervical dislocation. When performed correctly, blunt force trauma does result in immediate insensibility as measured by loss of auditory evoked potentials [8] and brainstem reflexes [12] in turkeys. Blunt force trauma is accepted under the condition that humane restraint is used, and the impact results in sufficient force and accurate placement to ensure immediate insensibility and death with a single attempt [1]. However, the skill required to humanely kill a bird using blunt force trauma, as well as the psychological impact of this method on the operator suggest that an alternative method may be more appropriate [3].

Percussive devices such as penetrating and non-penetrating captive bolts devices (NPCB) are alternative options that are not limited by the size of the bird, as with cervical dislocation. Non-penetrating captive bolt devices are intended to deliver sufficient kinetic energy to the head to result in immediate insensibility and brain death without penetrating the skin. NPCB devices address the potential biosecurity concerns that may arise with the use of penetrating captive bolt devices and are more reliable than blunt force trauma [12,34]. As with cervical dislocation and blunt force trauma, NPCB devices are considered to be acceptable with conditions for euthanasia of poultry. These conditions include proper restraint of the animal and adequate placement of the device on the head of the bird [1,3]. To date, there have been few published reports on non-penetrating captive bolt devices for use on turkeys: one using a prototype [12] and another in a laboratory setting looking at EEGs in only one age of turkey hens [35]. There are no previous reports on commercial models being tested on-farm by producers.

The objective of this study was to compare the efficacy of two commercially available non-penetrating captive bolt devices, the Turkey Euthanasia Device (TED) and the Zephyr-EXL (Bock Industries, Inc., Philipsburg, PA, USA), for on-farm euthanasia of turkeys at different stages of production. Efficacy was determined by evaluating ante-mortem signs of insensibility and clinical death, and post-mortem assessment of general trauma and brain damage. Based on previous reports, it was predicted that both of the NPCB devices would be highly efficient at producing immediate, fatal brain injury without recovery followed by death.

## 2. Materials and Methods

All procedures were approved by the University of Guelph Animal Care Committee (AUP #3321). The Zephyr-EXL and TED were evaluated on a total of 253 turkeys on ten different experimental days over a period of seven months. Birds were obtained from seven same-sex flocks that belonged to three large commercial turkey producers and one same-sex flock housed at a research facility. Twenty male toms were enrolled in this study following completion of a separate, terminal research project at the University of Guelph. All additional birds enrolled in this study were cull birds housed in same-sex commercial flocks requiring euthanasia as determined by farm protocols. These birds were selected either at placement on the farm, transfers between barns, or shipping for slaughter. Selection criteria included illness and injury resulting in birds not fit for transport.

### 2.1. Euthanasia Devices

**Figure 1:**
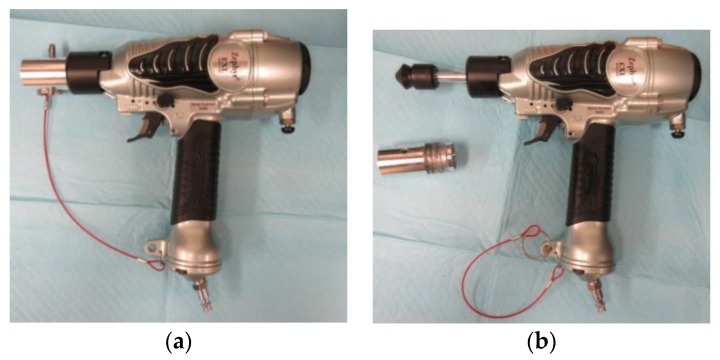
The non-penetrating captive bolt device (Zephyr-EXL) with safety pin inserted (**a**) and with subject adapter removed and bolt head exposed (**b**) [36].

The Zephyr-EXL (Figure 1) is a pneumatic-powered non-penetrating captive bolt device, consisting of a pneumatic nail gun fitted with a mushroom-shaped nylon head (diameter: 25 mm), and attached to a cylindrical metal bolt (diameter: 9.5 mm). The Zephyr-EXL is light-weight device (1.1 kg) that is attached to a standard air compressor or a portable CO_2_ power system. The specified energy expended is equivalent to 26 Joules with the bolt moving at 27 m/s when using an air compressor set to 120 PSI. The bolt protrudes 27.2 mm past the barrel during full extension. The Zephyr-EXL can be fired repeatedly in rapid succession by pressing the trigger. The performance of the Zephyr-EXL has been confirmed through tests with peak kinetic energy of 27.7 [24] and 24.4 [35] Joules when used at line pressure of 120 PSI.

**Figure 2:**
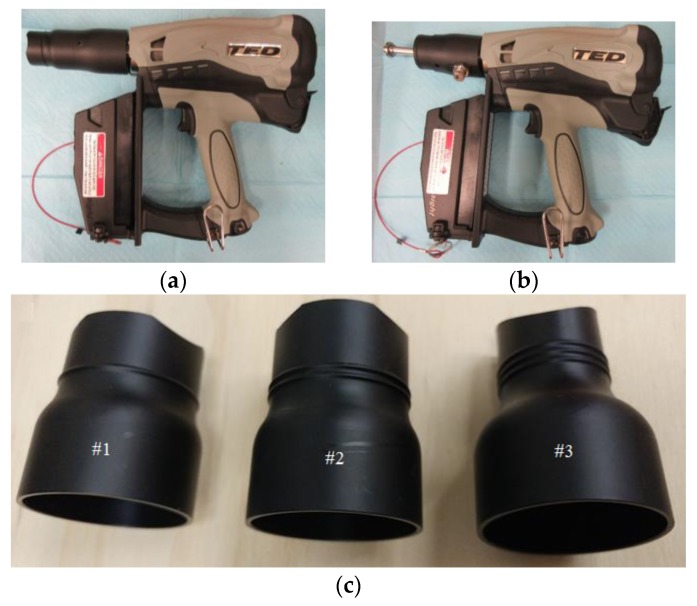
The non-penetrating captive bolt device (Turkey Euthanasia Device). (**a**) Device with adapter 1; (**b**) Device with adapter removed and bolt head exposed; (**c**) Subject adapters [36].

The TED is a fuel-powered non-penetrating captive bolt device (Figure 2). The TED consists of a modified compressed gas-powered nail gun (Hitachi NT65GS gas finish nailer) fitted with a flat metal head (diameter: 19.1 mm, length: 4 mm) and attached to a cylindrical metal bolt (9.5 mm). It is heavier than the Zephyr-EXL (1.8 kg vs. 1.1 kg) but does not require a separate power system (air compressor or gas bottle). The specified device energy expended is equivalent to 28 Joules with the bolt moving at 30 m/s [36]. The performance of the TED has been confirmed through tests, demonstrating peak kinetic energy of 28.4 Joules [35].

The TED has three adaptor options based on the weight of the bird (Figure 2c), as defined by the manufacturer. Adapter #1 has a maximum bolt travel distance of 11.7 mm and is designed for larger birds (>18 kg). Adapter #2 has a decreased bolt travel distance of 7.3 mm and is designed for birds 11 to 18 kg. Adapter #3 has the lowest bolt travel distance at 7.1 mm and is designed for smaller birds (<7 kg).

### 2.2. Euthanasia Procedure

A total of eight stock people and two researchers operated the devices during this study. Stock people were specific to each company; because of this, operator and company were confounded. Stock people were trained on-site before the start of each trial; each stock person was supervised by the researcher to ensure proper placement and use of each device. Any operator issues, such as incorrect placement, were recorded. Cull birds were separated from healthy birds and allocated to treatment groups during each day of the trial in an attempt to balance for sex and weight across device (Table 1).

**Figure 3:**
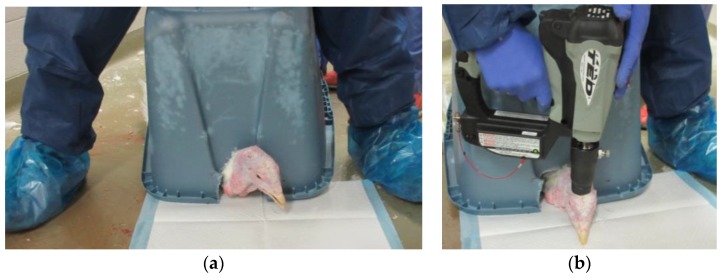
An example of the restraint method (**a**) and correct placement of the device (**b**) during the euthanasia trial.

Each turkey was placed on the barn floor in a sternal recumbent position with its keel on a solid, flat surface (cement pad or barn floor). Occasionally, shavings were placed on the ground for absorption of any bodily fluids. To restrain the birds and to protect both the stockperson and recorder from injury from wing flapping, a plastic bin was placed upside-down over top of the body of the bird to contain wing flapping and leg movements (Figure 3a). A slot was cut out from one end of the bin to allow the head to be accessed from outside of the box; this allowed for ease of device application and reflex monitoring. However, the restraint bin prevented assessment of rhythmic breathing immediately after device application. When wing flapping subsided in intensity and frequency, the bin was removed and the bird was placed in a dorsal recumbent position to monitor time to last movement and cardiac arrest.

The application of each device was carried out according to previous research recommendations with one discharge applied to the top of the head between the back of the eye and centre of the ear (Figure 3b) [12]. The Zephyr-EXL was set to 80 PSI for the 4–5 week-old turkeys, 100 PSI for the 10 week-old turkeys, and 120 PSI for the 15–20 week-old turkeys. The adapter used for the TED was based on manufacturer recommendations. Adapter #1 was used solely for toms in the 15–20 week-old group. Adapter #2 was used for all birds in the 10 week-old group and hens in the 15–20 week-old group. Adapter #3 was initially used for birds of both sexes in the 4–5 week-old group, but after experiencing some failures, males were subsequently switched to adapter #2.

### 2.3. Ante-Mortem Data Collection

Immediately after device application, turkeys were assessed for jaw tone, pupillary light reflex, and nictitating membrane reflex (Table 2). Reflexes were checked every 15 s until cardiac arrest. If eye reflexes were present at any time after a method was applied, the killing attempt was considered a failure. If pupillary light reflex or nictitating membrane reflex were present, a second discharge was applied immediately.

Time of last involuntary movement, either clonic or tonic convulsions, was also recorded (Table 2). Presence and duration of heartbeat were monitored through palpation and indirect auscultation. Cardiac arrest was estimated when no heartbeat could be palpated or auscultated with a stethoscope.

### 2.4. Post-Mortem Data Collection

External hemorrhage and skin lacerations were scored immediately after death. Turkey cadavers were then individually tagged, placed in a box for transportation, and taken to the University of Guelph for dissection and further macroscopic scoring. Distance from the farm locations was, on average, 100 km to the University of Guelph. Caution was taken during handling and transportation to prevent additional damage to the head and neck of the birds.

Gross dissection and macroscopic scoring of the skull fracture and brain hemorrhage were conducted on all turkeys successfully euthanized on the first attempt. External hemorrhage and skin laceration scores were recorded to determine external hemorrhage at the site of device application. Macroscopic scoring was conducted using a modified scale system adapted from previous euthanasia trials (Table 3) [19,22]. Subcutaneous hemorrhage (SC) was scored by removing the scalp and assessing the degree of hemorrhage on the dorsal surface of the skull from the back of the eyes to the base of the skull. The calvarium was then examined to determine the degree and extent of fracture(s) present. Following this, the calvarium and dura were removed and the degree of macroscopic subdural hemorrhage (SDH) was scored.

Following completion of gross dissection and scoring, a subset of brains was randomly selected for microscopic scoring and placed in 10% buffered formalin for at least ten days, prior to trimming. Initially, five brains per device per age group were scheduled for collection. However, due to the severe damage from the percussive devices, most brains were no longer intact and couldn’t be collected. As a result, only 24 brains from birds euthanized with the TED and 12 brains from birds euthanized with the Zephyr-EXL were still intact and available for collection. All trimming was completed by one individual to ensure consistency. Three sections of the brain (cerebrum, midbrain, and cerebellum) were sampled. Tissue sections were embedded in paraffin, sectioned, and stained with hematoxylin and eosin (Animal Health Laboratory, University of Guelph) prior to being examined and scored by a veterinary pathologist (PVT) blinded as to bird age, sex, and device type. The proportion of microscopic SDH and parenchymal hemorrhage (PH) was determined using a 0 to 4 scale and scored as no hemorrhage (0), minimal (<5%) hemorrhage (1), mild (5–10%) hemorrhage (2), moderate (>10–30%) hemorrhage (3) and marked (>30%) hemorrhage (4) [19,22,23,31].

All statistical analyses were performed using SAS 9.4 (SAS Institute Inc., Cary, NC, USA). Fisher’s Exact test was used to test the null hypothesis that the proportion of birds presenting ante-mortem measures following treatment was independent of killing method. Frequency tables were used to determine the number of birds presenting with ante-mortem measures. Mixed model analyses were used to test the fixed effects of the device, age, and their interaction on the duration of pupillary light reflex and nictitating membrane reflex, gasping, jaw tone, convulsions, and time at cardiac arrest; sex (nested within farm) and operator were included as random effects. Duration of gasping and jaw tone were log transformed to normalize the data. Raw means and standard errors are presented in the results. Generalized linear mixed models were used to test the effects of the device, age, and their interaction on macroscopic and microscopic damage using a multinomial distribution. Odds ratios were used to compare differences in the levels of fixed effects. Sex (nested within farm) and operator were included as random effects. For the microscopic scores, the effect of brain section on hemorrhage scores was first tested; because the section was not significant, the data from all sections were combined and the highest score from any brain section within a subject was used in further analyses to test for effects of the device, age, and their interaction.

Statistical significance was defined as *p* < 0.05 for all analyses.

## 3. Results

### 3.1. Ante-Mortem Evaluations

Euthanasia was considered to be successful in 119 out of 122 birds (97.5%) using the Zephyr-EXL, and 117 out of 131 turkeys (89.3%) for the TED, with turkeys presenting no pupillary light reflex or nictitating membrane reflex immediately after application. Application of the Zephyr-EXL resulted in three instances that were considered failures compared to the TED that resulted in 14 failures (*p* = 0.0145); the highest failure rate for the TED occurred in males of the 4–5 week-old group due to incorrect adapter selection (Table 4).

Reasons for device failures included previous head injury (*n* = 1) and undetected pressure drop with the air compressor (*n* = 2) for the Zephyr-EXL. Failures of the TED were a result of incorrect placement (*n* = 5), incorrect adapter selection (*n* = 5; Table 4), and previous head injury (*n* = 4). There were four failures when adapter 3 was used for 4–5 week-old males, but no failures at this age and sex with adapter 2. All 10 week-old turkeys and 15–20 week-old females were killed using adapter 2. All 15–20 week-old males were killed using adapter 1. Regarding previous head injuries, we subsequently identified ten mature turkey toms with similar lesions and tested both devices on these birds. For the birds with scabby lesions, 80% (4 out of 5) of the birds were unsuccessfully killed using the TED and 20% (1 out of 5) were unsuccessfully killed using the Zephyr-EXL.

Number of turkeys presenting eye reflexes, gasping, and jaw tone following application of the TED and Zephyr-EXL within the 3 age groups are given in Table 5. A device by age interaction was found for gasping, which was present in 16.2% of turkeys that were successfully killed using the TED and 0% of the turkeys killed using the Zephyr-EXL at 15–20 weeks of age (*p* = 0.0128). Of the turkeys that were gasping, the average duration of the gasping reflex was 28 ± 4.4 s with no difference between device (*p* = 0.463). Jaw tone did not differ between device (*p* = 0.902) but did show an age effect (*p* = 0.0003); no jaw tone was observed in 4–5 week-old birds compared to 15–20 weeks-old turkeys, in which jaw tone was noted in ~4% of birds. Of those birds with jaw tone, the average duration was 23 ± 0 s for 4–5 week-old turkeys, 15 ± 0 s for 10 week-old turkeys, and 39 ± 13.1 s for 15–20 week-old turkeys with no effect of age (*p* = 0.816).

Average time of last movement and cardiac arrest following application of the TED and Zephyr-EXL within 3 age groups are given in Table 6. The average time of last movement was 173 ± 6.5 s for the Zephyr-EXL and 188 ± 6.5 s for the TED (*p* = 0.102). Age affected latency to end time of movement (*p* = 0.0092) with latencies increasing with age. A device by age interaction was found for time to last movement with the TED having a longer latency than the Zephyr-EXL in 10 week-old turkeys (200 ± 9.6 s vs. 170 ± 6.6 s, respectively; *p* = 0.0015).

The average time to cardiac arrest was 214 ± 6.1 s for the Zephyr-EXL and 227 ± 6.3 s for the TED (*p* = 0.164). Age affected average time of cardiac arrest (*p* < 0.0001) with latencies increasing with age. A device by age interaction was found for cardiac arrest with the TED having a longer latency than the Zephyr-EXL in 10 week-old turkeys (246 ± 10.0 s vs. 198 ± 5.9 s; *p* = 0.0004).

**Figure 4:**
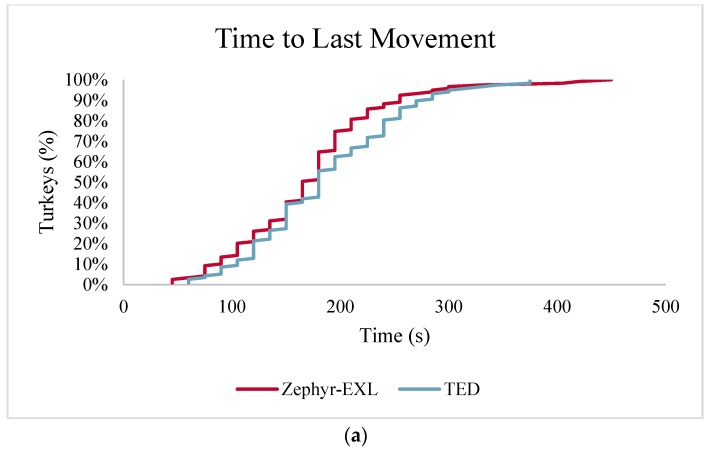

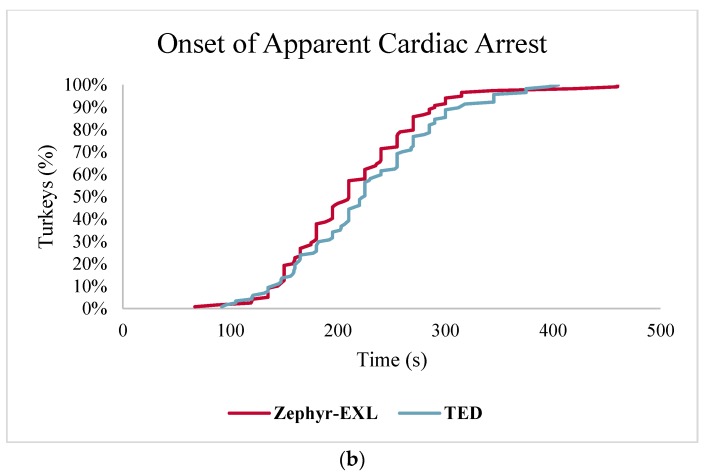
Cumulative frequency distribution for (**a**) time to last movement (s) and (**b**) time of apparent cardiac arrest (s) for turkeys killed with Zephyr-EXL or TED. Time point 0 indicates the time of application of the device.

The cumulative frequency distributions for the time to last movement and time of apparent cardiac arrest for turkeys euthanized with the TED and Zephyr-EXL for all age groups combined are plotted in Figure 4. Ninety-seven percent of turkeys euthanized with either the Zephyr-EXL or TED achieved last movement within 5 min (Figure 4a). Turkeys euthanized with the Zephyr-EXL achieved cardiac arrest slightly earlier with 94% of turkeys achieving cardiac arrest within 5 min; whereas 89% of turkeys euthanized with the TED achieved cardiac arrest within 5 min (Figure 4b).

### 3.2. Post-Mortem Macroscopic Assessment

The frequency of macroscopic scores by device and age are presented in Table 7. The severity of external hemorrhage and skin laceration (EHSL) was not affected by device (*p* = 0.930). All turkeys had penetrating skull fractures, with no difference between device (*p* = 1.000) or age (*p* = 1.000). Subcutaneous hemorrhage was affected by age (*p* < 0.0001) as there was an 18.0 times greater chance of higher scores with 4–5 week-old turkeys compared to 15–20 week-old turkeys and 14.0 times greater chance of a higher score in the 10 week-old turkeys.

Subdural hemorrhage scores were affected by device, age and their interaction. The TED had a 5.6 times greater chance of lower score compared to the Zephyr-EXL (*p* < 0.0001). Older turkeys (15–20 weeks of age) had a 2.8 times greater chance of a lower score compared to the 4–5 week-old turkeys, and a 2.4 times greater chance of a lower score compared to the 10 week-old turkeys. For turkeys euthanized with the Zephyr-EXL, there was 6.4 times greater chance of a lower score at 15–20 weeks of age compared to 4–5 week of age, and 5.8 times greater chance of a lower score compared to the 10 week-old turkeys. No age by device interaction was found for turkeys killed with the TED (*p* = 0.795).

### 3.3. Post-Mortem Microscopic Evaluation

Scores from microscopic examination of the different sections of the brain were compared. There was no effect of the device on scores for the different brain sections for either microscopic SDH (*p* = 0.803) or PH (*p* = 0.497). Therefore, data from the different brain sections were combined and the highest score for each brain was used in final analyses. The frequency distributions of microscopic scores are presented by device in Table 8 and by age in Table 9.

The small and unequal sample sizes for microscopic scoring did not allow for evaluation of device by age interactions, and values for device and age are presented separately. Subdural or parenchymal hemorrhage was present in at least one section of every brain scored. The highest microscopic SDH score from each brain indicated moderate to severe hemorrhage (scores 2–4) in 92% of turkeys for the Zephyr-EXL and 96% of turkeys for the TED. The highest PH score from each brain indicated moderate to severe hemorrhage (scores 2–4) in 83% of turkeys for both the Zephyr-EXL and TED. No differences were found between devices for either the subdural (*p* = 0.844) or parenchymal (*p* = 0.301) hemorrhage within the brain. There was no effect of age on any of the scores for microscopic brain hemorrhage (Table 9): SDH (*p* = 0.787), brain PH (*p* = 0.253).

## 4. Discussion

This trial compared the efficacy of two commercial non-penetrating captive bolt devices, the TED and Zephyr-EXL, for humane, on-farm killing of turkeys across three ages. Brainstem reflexes—pupillary and nictitating membrane—have been previously used as practical measures for determining insensibility and brain death in poultry [13,37]. The reflex responses of turkeys under different clinical states of sensibility (awake, semi-awake under sedation, insensible in a deep hypnotic state under general anesthesia, and dead following barbiturate overdose) were previously examined and validated using EEG spectral analysis [7]. In that study, the nictitating membrane reflex was sporadically present in a few birds otherwise determined to be dead, and the authors concluded that this reflex response is a conservative measure that can be used to confirm brain death in poultry; jaw tone was lost under anesthesia but pupillary reflex was present until death [7]. As such, immediate loss of jaw tone, nictitating eye reflex, and pupillary light reflex were chosen as highly conservative criteria for successful euthanasia in this study. While one study reported that the pupillary light reflex is difficult to assess due to lighting condition and bird convulsions, it was used in this study without issue [12].

One benefit of using these NPCB devices is the lack of direct restraint required to apply the device. In theory, the lack of restraint would help reduce additional stressors and may prevent additional injuries to the bird. However, to ensure appropriate positioning of the device, most guidelines recommend some form of restraint [3]. To maintain consistency of application and for measurement in this study, turkeys were placed on the barn floor in sternal recumbency with large birds restrained using a plastic bin and small birds restrained by hand. Research in humans has shown spontaneous movements in brain-dead patients that can occur as a result of stimulation [38]. The uniformity in restraint between both devices was key to ensuring any changes as a result of restraint were consistent with every bird. The restraint method used in this study provided complete access to the head of the bird to fully assess eye reflexes, gasping, and jaw tone. However, the same restraint method limited monitoring of rhythmic breathing. The cost, ease of use, and minimal stress that occurs when catching and restraining a bird, as well as safety for the operator make this method for restraint of large turkeys highly practical in the field.

Results from ante-mortem measures demonstrated that both devices are acceptable methods for turkey euthanasia, however, the Zephyr-EXL (98%) did have a higher success rate compared to the TED (89%). The Zephyr-EXL and TED were both designed specifically as euthanasia devices with considerable development and testing of prototypes. The absence of pupillary and nictitating eye reflexes are consistent with some studies of similar NPCB devices used for euthanasia, in which insensibility and brain death were considered to be immediate based on a lack of brainstem reflexes [12,22,23,31] or visual evoked potential [5,24]. DEFRA (Department of Environment, Food & Rural Affairs) created a pneumatic stunner with variable bolt heads that showed promising results. A similar device was then developed for stunning and killing broilers that successfully induced brain death with correct placement and angle; however, this device is not commercially available [39].

A few studies have shown poor success rates when using some other percussive devices for on-farm euthanasia. For example, a NPCB device used on 7 to 8-week-old piglets was completely unsuccessful with all six piglets surviving due to insufficient brain damage [21]. Similarly, 17 of 60 chickens were unsuccessfully killed using a modified penetrating captive bolt device (Rabbit Zinger^TM^) [13]. Differences in device success have been linked to sufficient force [12,21,22], correct placement [12,21], and proper restraint [23]. The development of percussive bolt designs and subsequent testing of these devices emphasizes the importance of device and species differences that can occur and highlight that species-specific euthanasia devices be used to assure optimal performance and success for on-farm euthanasia.

The failures of both devices in turkeys with a previous head injury or head lesions are an interesting finding that warrants future research. Head injuries represent a major cause for culling, especially in tom turkeys, where injurious pecking directed at the head is a common behaviour problem and welfare issue [40,41]. Head injuries in the present study were identified as birds with dark, thickened skin covering the bird’s head and neck. Dry, necrotic tissue was found subcutaneously in these animals. It is possible that this abnormal skin may provide a cushioning or deflecting effect, somehow reducing the impact of the NPCB device to the brain. Subsequent to this study, we identified ten mature turkey toms with similar lesions and tested both devices on these birds. For the birds with scabby lesions, 80% (4 out of 5) of the birds were unsuccessfully killed using the TED and 20% (1 out of 5) were unsuccessfully killed using the Zephyr-EXL. Therefore, it is important to determine whether these devices can function properly when used on birds with severe head injuries, or whether a different placement or bolt head should be adopted.

Regarding loss of pressure with the air compressor when using the Zephyr-EXL, this occurred within the first couple of discharges. To avoid this, we recommend that the operator check the compressor pressure prior to restraining the animal for discharge. Routinely checking and maintaining recommended air pressure during device operation will prevent this error from occurring.

Time to last movement has been suggested as an indicator of the time of brain death in birds because it had previously been observed to occur at the same time as cessation of brain activity when birds have been killed using CO_2_ [18,42] or high expansion foam [16]. In this study brain death occurred immediately after application of each treatment, as measured through pupillary and nictitating eye reflexes and as confirmed by the severity of both macroscopic and microscopic indicators of traumatic brain injury. Time to last movement occurred much later than the loss of eye reflexes and ended shortly before cardiac arrest. This suggests that duration of involuntary movements may be dependent on the specific mode used for inducing death (e.g., inhaled gas versus physical methods) [7,37,43,44]. It also suggests that birds should be monitored until the time to last movement in order to confirmed death before disposal.

Gasping or non-rhythmic agonal breathing that occurs as a result of a spinal reflex [24], was noted in several older turkeys euthanized with the TED in this study. However, the duration of gasping was relatively short (28 ± 4.4 s) and occurred following signs of brain death. This suggests that, in the present study, gasping was unlikely to be associated with sensibility or distress. This supports previous research in which gasping was not observed in turkeys killed with percussive devices [12]. However, in that same study gasping was observed in turkeys killed by manual and mechanical cervical dislocation at the same time that brainstem reflexes were present [12]. This suggests that gasping can be indicative of a sign of respiratory distress when occurring in sensible animals measured through brainstem reflexes.

Researchers found that more skull fractures were present in pre-weaned kits than adults in an on-farm euthanasia trial in rabbits with the Zephyr-E [31]. Through assessment of external hemorrhage and skull fractures, appropriate pressures were determined based on the skull development and thickness and the researchers recommended altering pressure settings for NPCB devices rather than applying maximum pressure to all animals to control for excessive external hemorrhage and related biosecurity and aesthetic concerns [31]. If not previously validated using cadavers, as in this study [31], ad hoc adjustments of air pressure may be problematic. In the current study, pressure levels were reduced for smaller birds with 80 PSI used in 4–5 week-old turkeys. Pressure levels were lowered to 100 PSI for 10 week-old turkeys and maintained at 120 PSI for all 15–20 week-old turkeys. Compressor pressure reduction helped reduce external hemorrhage while still inducing significant brain injury and death. The adjustment of air pressure when using the Zephyr-EXL is comparable to selecting the appropriate bolt adapter when using the TED. Results from the present study confirm findings that pressure settings can be altered based on the age or weight of the bird to improve aesthetics and minimize biosecurity risks [31]. When choosing an appropriate method of euthanasia, there is a fine balance between effectiveness and aesthetics that must be taken into consideration. Improving the aesthetics while maintaining high success rates will allow for this device to be adopted on farms and accepted by both producers and observers of the procedure. Future studies should compare adapter selection and pressure settings within more specific weight classes of turkeys to minimize external hemorrhage thereby reducing the biosecurity risk and improving aesthetics.

Both the Zephyr-EXL (93%) and TED (92%) resulted in high external hemorrhage scores, a consideration for observer esthetics. A weakness with the macroscopic scoring system used for external hemorrhage in this study was the broad category classifications. A hemorrhage score of two was given if the skin was lacerated and blood was present. However, there was no differentiation between a few drops and a large pool of blood. Future studies using this type of scoring should refine the scoring system used to better differentiate between the volumes of blood present at the site of impact. In addition, producers expressed concern about the high external hemorrhage because they believed the blood may trigger aggression among male turkeys if birds were to be euthanized within the barn.

Microscopic subdural and parenchymal hemorrhage scores were moderate to severe for both devices. Hemorrhage within these regions is indicative of focal to regional brain injury in humans, depending upon the extent and severity [45]. These microscopic hemorrhage scores confirm that both the TED and Zephyr-EXL caused substantial damage to the target regions deep within the brain that are responsible for sensibility and vital function.

Lack of operator experience was consistent across farms, with the experience of the researcher being the exception. One operator had experience with the original version of the TED but no experience with the newer model or with the Zephyr-EXL. All remaining operators had no previous experience with either device. The very high degree of device success indicates that minimal training is required; however, practice on cadavers may be beneficial to aid in the operator’s confidence with device placements and should be encouraged. Anecdotal evidence acquired from farm staff feedback showed device preference for the TED over the Zephyr-EXL, despite the higher success rate of the Zephyr-EXL; suggesting that the portability and convenience to the operator may take precedence over the efficacy of the device.

## 5. Conclusions

In conclusion, the Zephyr-EXL and TED both meet the strict criteria for success and are effective and reliable methods for single-step on-farm euthanasia of turkeys when the appropriate positioning and adapter or compressor pressure are used. Both devices caused similar skull fractures and microscopic damage to the brain with a marginally higher success rate with the Zephyr-EXL. Post-mortem assessment results strongly correlated with the ante-mortem assessments of insensibility and brain death. The TED has the advantage of portability within the barn but may be restricted from use in turkeys with severe head injuries. This study provided quantitative data for two commercially available NPCB devices for euthanasia of turkeys and can be used in the development of recommendations for on-farm humane killing recommendations.

## Figures and Tables

**Table 1:** Description of sample sizes and device distribution across age, sex, farm, and operator.

Age (Weeks)	Average Weight (kg)	Sex	Number of Farms	Number of Operators	Device	Sample Size
4–5	1.3	Female	2	1	TED	22
					Zephyr-EXL	21
	2.0	Male	1	3	TED	29
					Zephyr-EXL	20
10	4.0	Female	1	1	TED	19
					Zephyr-EXL	19
	8.0	Male	1	3	TED	21
					Zephyr-EXL	20
15–20	10.0	Female	1	1	TED	20
					Zephyr-EXL	20
	14.9	Male	3	5	TED	20
					Zephyr-EXL	22

**Table 2:** List of reflexes, description, and procedure use, recorded in order of observation after application of each killing method.

Measure	Description	Procedure
Pupillary	Constriction of the pupil in response to light	Light from a medical penlight was directed into the eye
Nictitating	Transient closure of the nictitating membrane in response to mechanical stimulation	The medial canthus of the eye was lightly touched with a fingertip
Gasping	Deep breathing with the mouth open wide	Visual observation for an open beak with irregular deep breaths
Jaw Tone	Resistance due to downward pressure applied to the jaw	Gentle pressure was applied to the lower jaw with a finger
Tonic	Muscle rigidity with final paddling motions with the legs and wings stretched	The time of cessation of all movement was recorded. Convulsions ceased when the limbs were relaxed
Clonic	Episodes of wing-flapping	See Tonic.
Cardiac Arrest	Cessation of heart beat	Palpation of brachial and femoral arteries, and indirect auscultation

**Table 3:** Macroscopic scoring criteria for trauma, fracture and hemorrhage, modified from [19,22].

Score	Skull Fracture	External Hemorrhage and Skin Laceration	Subdural Hemorrhage
0	No fracture, intact skull	No laceration the skin	No hemorrhage
1	Depression fracture *	Laceration of the skin with no external hemorrhage	<25% of surface area covered
2	Penetrating fracture/no imbedded fragments	Laceration of the skin with external hemorrhage	26–50% of surface area covered
3	Penetrating fracture/with imbedded fragments	N/A	51–75% of surface area covered
4	N/A	N/A	75–100% of surface area covered

* Depression fractures are incomplete fractures and penetrating fractures are complete fractures.

**Table 4:** Number of failures by device type, bird age, sex, and adapter selection.

Age (Weeks)	Device	Sex	*N*	Number of Failures	Adapter
4–5	TED	Female	22	2	3
		Male	29	7	2,3
	Zephyr-EXL	Female	21	0	N/A
		Male	20	1	N/A
10	TED	Female	19	1	2, 3
		Male	21	1	2
	Zephyr-EXL	Female	19	0	N/A
		Male	20	0	N/A
15–20	TED	Female	20	1	2
		Male	20	2	1
	Zephyr-EXL	Female	20	0	N/A
		Male	22	2	N/A

**Table 5:** Number of birds presenting with reflexes and involuntary behaviours following application of the Turkey Euthanasia Device (TED) and Zephyr-EXL.

Reflex	Device	*N*	Age (Weeks)			Total	*p*-Value		
			4–5	10	15–20		Device	Age	Device × Age
Pupillary *	TED	131	9	2	3	14 ^c^	**0.0145**	0.139	0.206
	Zephyr-EXL	122	1	0	2	3 ^d^			
Nictitating *	TED	131	9	2	3	14 ^c^	**0.0145**	0.139	0.206
	Zephyr-EXL	122	1	0	2	3 ^d^			
Gasping	TED	117	2 ^a^	0 ^a^	6 ^c,b^	8 ^c^	**0.0022**	**0.0128**	**0.0128**
	Zephyr-EXL	119	0	0	0 ^d^	0 ^d^			
Jaw Tone	TED	117	0 ^a^	0 ^a^	5 ^b^	5	0.902	**0.0003**	0.6300
	Zephyr-EXL	119	0	1	4	5			

* Scores includes turkeys that were deemed failures. ^a,b^ Indicates differences observed within age by device. ^c,d^ Indicates differences observed within age. Bold is used to indicate significance.

**Table 6:** Average end time of last movement and cardiac arrest for turkeys killed using either device (TED and Zephyr-EXL) across three age groups (4–5 weeks, 10 weeks, and 15–20 weeks), in addition to the mean value of the device by age interaction.

Device				*p-*Value		
Age (Weeks)	TED	Zephyr-EXL	Mean for Age	Device	Age	Device × Age
**Time to Last Movement**						
4–5	186 ± 11.9	139 ± 13.3	163 ± 9.2 ^c^	0.102	**0.0092**	**0.0015**
10	200 ± 9.6 ^a^	170 ± 6.6 ^b^	185 ± 6.04 ^d^			
15–20	178 ± 11.7	209 ± 10.1	194 ± 7.9 ^d^			
Overall	188 ± 6.5	173 ± 6.5				
**Cardiac Arrest**						
4–5	209 ± 11.3 ^c^	185 ± 12.1 ^c^	197 ± 8.3 ^c^	0.164	**<0.0001**	**0.0004**
10	246 ± 10.0 ^a,d^	198 ± 5.9 ^b,c^	222 ± 6.4 ^d^			
15–20	228 ± 10.9	260 ± 8.4 ^d^	244 ± 7.0 ^d^			
Overall	227 ± 6.3	214 ± 6.1				

^a,b^ Indicates differences observed within age. ^c,d^ Indicates differences observed within age difference by device. Bold is used to indicate significance.

**Table 7:** Distribution of macroscopic hemorrhage scores following application of the Turkey Euthanasia Device (TED) and Zephyr-EXL.

Variable *	Device	Age	*N*	Score					*p*-Value		
				0	1	2	3	4	Device	Age	Device × Age
EHSL	TED	4–5	42	3	1	38	N/A	N/A	0.930	0.455	**0.0018**
		10	38	5	4	29					
		15–20	37	3	9	25					
		Total	117	11	14	92					
	Zephyr-EXL	4–5	40	11	3	26					
		10	39	7	2	30					
		15–20	40	1	2	37					
		Total	119	19	7	93					
Skull Fx	TED	4–5	42	0	0	0	42	N/A	1.000	1.00	1.000
		10	38	0	0	1	37				
		15–20	37	0	0	4	33				
		Total	117	0	0	5	112				
	Zephyr-EXL	4–5	40	0	0	0	40				
		10	39	0	0	2	37				
		15–20	40	0	0	0	40				
		Total	119	0	0	2	117				
SC	TED	4–5	42	0	0	0	3	39	0.143	**<0.0001**	0.851
		10	38	0	1	1	2	34			
		15–20	37	1	10	5	7	14			
		Total	117	1	11	6	12	87			
	Zephyr-EXL	4–5	40	0	0	2	0	38			
		10	39	0	1	0	1	37			
		15–20	40	0	4	6	7	23			
		Total	119	0	5	8	8	98			
SDH	TED	4–5	42	0	3	8	17	14	**<0.0001**	**0.0037**	**0.0009**
		10	38	0	2	9	17	10			
		15–20	37	0	1	6	20	10			
		Total	117	0	6	23	54	34			
	Zephyr-EXL	4–5	40	0	0	1	5	34			
		10	39	0	1	2	3	33			
		15–20	39	0	2	10	7	20			
		Total	118	0	3	13	15	87			

* External hemorrhage and skin laceration (EHSL), skull fracture (Skull Fx), subcutaneous hemorrhage (SC), and subdural hemorrhage (SDH). Bold is used to indicate significance.

**Table 8:** Distribution of microscopic hemorrhage scores following application of the Turkey Euthanasia Device (TED) and Zephyr-EXL.

Variable	*N*	Device	Score					*p*-Value *
			0	1	2	3	4	Device
SDH	24	TED	0	1	7	16	0	0.844
	12	Zephyr-EXL	0	1	2	9	0	
PH	24	TED	0	4	7	13	0	0.301
	12	Zephyr-EXL	1	1	4	6	0	

* *p*-values indicates the probability of hemorrhage score variation as a result of device differences for subdural (SDH) and parenchymal (PH) hemorrhage.

**Table 9:** Distribution of microscopic hemorrhage scores by age.

Variable	Age (Weeks)	*N*	Score					*p*-Value *
			0	1	2	3	4	Age
SDH	4–5	5	0	0	0	0	5	0.787
	10	12	0	0	1	3	8	
	15–20	19	0	0	1	6	12	
PH	4–5	5	0	0	1	2	2	0.253
	10	12	1	0	3	3	5	
	15–20	19	0	0	1	6	12	

* *p*-values indicates the probability of hemorrhage score variation as a result of age differences for subdural (SDH) and parenchymal (PH) hemorrhage.
